# Multiscale systems modelling of communication networks in brain metastasis

**DOI:** 10.1038/s12276-026-01774-4

**Published:** 2026-07-06

**Authors:** Ju Young Ahn, Wenjuan Dong, Stephen TC Wong, Hong Zhao

**Affiliations:** 1 Systems Medicine and Bioengineering Department, Houston Methodist Neal Cancer Center, Houston, TX 77030 USA; 2 Department of Biomedical Engineering, The Texas A&M University, College Station, TX 77843 USA; 3 College of Medicine, The Texas A&M University, Bryan, TX 77807 USA; 4 Department of Neurology, Houston Methodist Hospital, Houston, TX 77030 USA; 5 Department of Medicine, Weill Cornell Medicine, New York, NY 10065 USA

**Keywords:** CNS cancer, Computational models

## Abstract

Brain metastasis remains a major clinical challenge because intracranial progression and therapeutic resistance arise not only from tumour-intrinsic programmes but also from coordinated communication across molecular, cellular, spatial and systemic scales. In this Review, we integrate findings from spatial transcriptomics/proteomics, single-cell and multimodal omics, and computational systems modelling to frame brain metastasis as a multiscale communication disorder in which immune, glial, vascular and systemic networks collectively shape tumour-permissive ecosystems. We discuss how network-based analyses identify communication hubs that can be translated into therapeutic hypotheses and provide a rationale to guide drug repurposing and rational combination strategies targeting tumour–microenvironment interactions. We further examine how multiscale modelling and artificial-intelligence-enabled integration of spatial and molecular data may enable patient stratification, response prediction, and adaptive trial designs, while highlighting key barriers, including data harmonization, spatial resolution limits, model interpretability and clinical deployment constraints. Reframing brain metastasis as a multiscale communication disorder provides a unifying conceptual and methodological foundation for therapies that disrupt metastatic ecosystems and improve durable intracranial disease control.

## Brain Metastasis as a Multiscale Communication Disorder

Brain metastases, a severe complication of advanced cancer, significantly impact patient survival and quality of life. Despite advances in cancer therapies, brain metastases remain challenging owing to the brain’s unique microenvironment, which fosters therapeutic resistance. This review highlights the role of the brain’s microenvironment, including glial cells and immune interactions, in shaping metastatic progression and drug resistance. The authors propose a multiscale systems modelling framework to understand these interactions across molecular, cellular and systemic levels. They emphasize the importance of spatially resolved technologies and network-based therapeutic strategies to identify communication hubs that sustain tumour-permissive states. The study suggests that targeting these communication networks, rather than isolated pathways, could offer new therapeutic opportunities. Future research should focus on integrating these insights into clinically actionable models to improve treatment outcomes for patients with brain metastasis.

This summary was initially drafted using artificial intelligence, then revised and fact-checked by the author.

## Introduction

Brain metastases represent one of the most lethal complications of advanced cancer, frequently determining quality of life and survival even when extracranial disease is controlled. Despite major advances in targeted agents and immunotherapies, median survival is still often measured in months, and up to 30–40% of patients with metastatic disease develop brain involvement^1–3^. Although molecular profiling has revealed recurrent oncogenic programmes that support metastatic fitness^4,5^, therapeutic resistance in the brain has nonetheless proven remarkably persistent, limiting the efficacy of tumour-intrinsic targeting strategies^6–10^. Increasingly, evidence points instead to the brain microenvironment as a dominant regulator of metastatic progression and treatment response, shaping both colonization and the emergence of drug-tolerant states^11,12^.

Unlike extracranial tissues, the central nervous system is characterized by restricted immune surveillance, limited therapeutic access imposed by the blood–brain barrier, and a cellular ecosystem dominated by glial cells^13^. Once metastatic tumour cells breach the blood–brain barrier, their survival in the brain depends not only on intrinsic genetic adaptations but also on their ability to engage and reprogramme local glial networks^14^. Accumulating evidence indicates that astrocytes, microglia and oligodendrocyte-lineage cells actively participate in shaping metastatic niches by modulating immune surveillance, metabolic support and stress signalling^15–17^. These interactions are neither static nor uniform; rather, they are spatially organized, dynamically evolving and highly context dependent^11,15,16,18,19^.

Beyond canonical tumour-immune crosstalk, non-canonical modulators of tumour–brain communication has further expanded the complexity of the metastatic niche. Latent neurotropic viral infections, such as cytomegalovirus (CMV), can reshape immune and glial signalling programmes through persistent, low-level reactivation, potentially amplifying immunosuppression and promoting tumour-permissive states^20,21^. Expanding on this concept, our recent work indicates that CMV viral activity rewires immune–glial communication networks in brain metastases, providing mechanistic support for viral modulation of metastatic progression^22^. Systemic factors, including chronic stress and neurotransmitter signalling, further influence glial and immune behaviour, effectively coupling neuroendocrine physiology to metastatic progression^23–25^. Together, these observations support a unifying view: brain metastasis emerges not from isolated oncogenic pathways but from the coordinated dysregulation of multiscale communication networks spanning immune, glial, viral and neuroendocrine systems.

Capturing the behaviour of this system requires approaches that preserve spatial context and temporal dynamics. Bulk omics obscure spatial context, single-cell profiling without spatial resolution can miss emergent tissue-level organization and static snapshots fail to represent the temporal evolution of metastatic niches. As a result, critical communication hubs and feedback loops that sustain therapeutic resistance remain hidden, and mechanistically actionable targets can be difficult to prioritize from descriptive datasets alone.

In this review, we frame brain metastasis as a multiscale communication disorder and propose a multiscale systems modelling framework that integrates molecular, cellular, tissue and systemic levels to examine how immune, glial and viral signalling networks interact to shape metastatic behaviour. We further discuss emerging technologies that enable spatially resolved, multimodal and longitudinal modelling of these networks, and explore how network-based therapeutic strategies and drug repurposing approaches may offer new opportunities to overcome resistance in the metastatic brain while emphasizing the need for causal validation, cross-cohort reproducibility and clinically deployable readouts for translation.

## Multiscale communication framework in brain metastasis

**Fig. 1:**
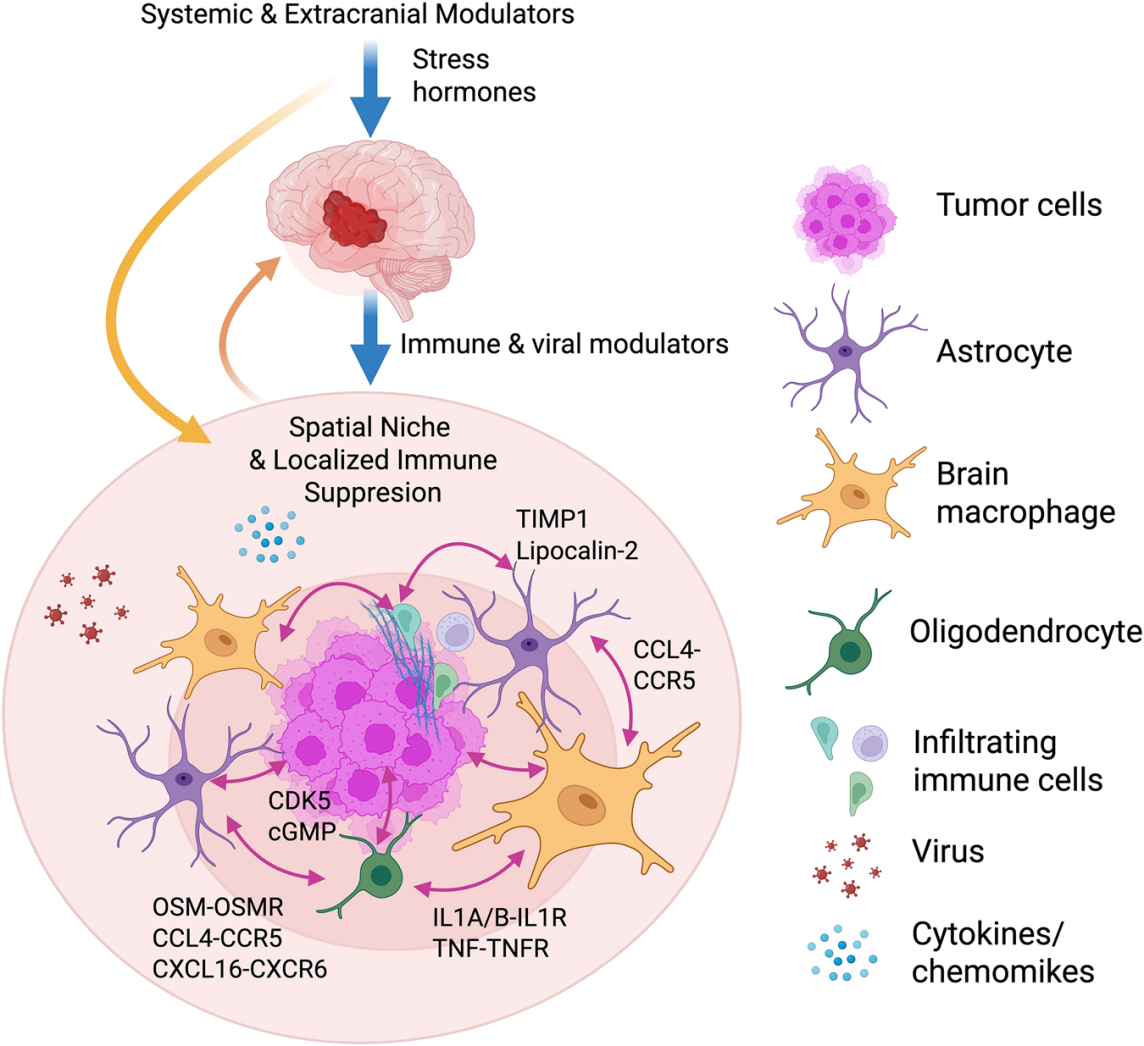
Multiscale communication networks in brain metastasis. Brain metastasis develops within spatially organized communication niches formed by interactions between metastatic tumour cells and brain-resident and infiltrating cell populations. Astrocytes, microglia, immune cells and stromal components engage in bidirectional signalling through cytokine, chemokine and receptor-mediated pathways, including CCL4-CCR5, OSM-OSMR, IL1-IL1R and TNF-TNFR signalling, as well as astrocyte-derived factors such as TIMP1 and lipocalin-2. Gap junction signalling and astrocyte-induced Cdk5 activation further promote immune evasion and tumour survival. These interactions concentrate at the tumour–brain interface and other microdomains where spatial proximity reinforces feedback loops that stabilize tumour-supportive ecosystems. Extracranial modulators, including stress-related neuroendocrine inputs and viral-associated signals, tune these circuits across scales and contribute to disease progression and therapeutic resistance. Created using BioRender.

Brain metastasis cannot be adequately explained by tumour-intrinsic signalling alone. Instead, it emerges from coordinated communication across multiple biological scales, spanning molecular interactions, cellular state transitions, tissue-level organization, and systemic physiological inputs^26^. Figure 1 illustrates this multiscale communication framework, in which metastatic tumour cells are embedded within reciprocal (bidirectional), dynamically interacting immune, glial, viral and neuroendocrine networks that collectively shape disease progression and therapeutic resistance. This framework also makes a practical prediction: clinically durable control will require interventions that disrupt reinforcing cross-scale feedback, not only single-node tumour pathways.

### Molecular communication: shared signals, context-dependent outcomes

On the molecular scale, communication is mediated by ligand–receptor interactions, cytokines, chemokines, neurotransmitters and stress-associated signalling pathways that converge on shared downstream programmes regulating immune suppression, metabolic adaptation and stress responses. Direct experimental evidence has shown that tumour cells establish functional communication with astrocytes through connexin-based gap junctions, enabling transfer of second messengers such as cGAMP that activate inflammatory signalling in astrocytes and promote metastatic survival^27^. Additional studies have demonstrated that astrocytes can promote immune evasion by suppressing antigen presentation pathways and modulating infiltrating immune cells, including STAT3-dependent astrocyte activation, TIMP1-mediated local immunosuppression and lipocalin-2-mediated reciprocal interactions between innate immune cells and astrocytes^12,28–30^. Chemokine signalling represents an additional molecular mechanism coordinating intercellular communication within the metastatic niche. In particular, CCL4-CCR5 signalling has been shown to regulate tumour-microenvironment interactions and downstream immune and survival pathways, highlighting how chemokine gradients contribute to metastatic adaptation within the brain microenvironment^16^. Complementary work from the Joyce laboratory has shown that stromal and endothelial compartments also contribute to immune regulation within brain tumours^31,32^, highlighting that molecular signalling control is distributed across multiple non-tumour cell populations rather than confined to tumour–immune interactions alone.

Importantly, identical molecular signals may produce distinct biological outcomes depending on cellular identity, activation state and spatial localization within the brain microenvironment. A key rigour point is that inferred ligand–receptor “activity” is not equivalent to functional signalling; it requires orthogonal validation (e.g. protein-level detection, pathway activation, perturbation or blockade) and attention to confounders such as cell-state composition and spatial proximity. Systems immunology analyses further demonstrate that diverse signalling inputs converge onto shared transcriptional and immune regulatory programmes, emphasizing that network-level integration and pathway redundancy rather than individual pathways govern metastatic adaptation^14^.

### Cellular communication: distributed control across the metastatic ecosystem

On the cellular scale, distinct populations within the brain metastatic microenvironment adopt specialized yet interdependent roles that collectively regulate tumour behaviour^33^. Astrocytes respond rapidly to metastatic invasion and function as intermediaries linking tumour-derived signals to immune modulation and tissue remodelling, consistent with early observations that reactive glia promote tumour-cell colonization and survival^34–36^. Microglia and infiltrating macrophages exhibit substantial phenotypic plasticity, with transcriptional profiling studies revealing disease-specific activation states shaped by local microenvironmental signals rather than fixed lineage programmes^17,37^. Work from the Joyce laboratory further demonstrated that immune-cell activation states within brain tumours are instructed by local tissue context^38,39^, reinforcing the concept that immune phenotypes emerge from niche-derived signals rather than intrinsic immune programming. In addition to immune populations, endothelial and stromal compartments contribute to niche stabilization through immune-regulatory signalling^37,40–42^, whereas oligodendrocyte lineage cells increasingly appear to participate in tissue stress responses and glia–glia communication influencing neural integrity^43^. Systems-level analyses integrating spatial and single-cell datasets support a model in which tumour progression emerges from coordinated cellular-state transitions across interacting cell populations rather than from dominant influence by a single cell type^14^. Consistent with this framework, chemokine-mediated signalling through the CCL4–CCR5 axis has been shown to facilitate communication between tumour cells and brain-resident stromal populations^16^, reinforcing tumour-supportive cellular states through indirect immune and metabolic regulation.

### Tissue architecture: spatial niches as communication amplifiers

On the tissue scale, communication becomes spatially organized into structured niches rather than being uniformly distributed across metastatic lesions. Imaging and spatial profiling studies have demonstrated that metastatic outgrowth preferentially occurs along existing brain vasculature and at tumour–brain interfaces^44^, where localized immune suppression, altered metabolic states and disrupted neural homeostasis create permissive environments for tumour persistence^40,45^. More recent spatial transcriptomic and imaging-based analyses have shown that signalling interactions concentrate within defined microdomains enriched for tumour cells, astrocytes and immune populations^37,44,46^, indicating that spatial proximity amplifies signalling feedback loops and stabilizes niche-specific biological states. Systems immunology approaches integrating spatial and computational modelling further demonstrate that these tissue-level interactions generate emergent properties that cannot be inferred from dissociated single-cell measurements alone^14^.

Spatial analyses of chemokine signalling further suggest that CCR5-associated signalling pathways are enriched in tumour-adjacent regions, supporting the concept that chemokine-mediated communication contributes to the stabilization of spatially organized metastatic niches^16^. Importantly, tumours occupying distinct anatomical regions of the brain such as cortex versus thalamus areas display different signalling environments despite similar tumour genotypes^16^, emphasizing that metastatic behaviour arises from tissue-level organization and local context. This motivates explicit modelling of spatial neighbourhoods, boundary zones and vascular-adjacent compartments, and it argues for cross-platform replication (multiple spatial technologies) to ensure the robustness of niche definitions.

### Systemic and extracranial modulators: embedding brain metastasis in whole-organism physiology

Overlaying local tumour-niche interactions are modulatory inputs from latent or persistent neurotropic viruses and systemic physiology, as represented in the outer layers of Fig. 1. Beyond CMV, multiple neurotropic viruses (including herpesviruses and polyomaviruses) have been reported in brain-tumour tissues or brain-resident cellular compartments, raising the possibility that chronic low-level viral activity or viral gene expression can reshape immune tone and glial activation without requiring overt lytic infection or direct oncogenic transformation^47–49^. Recent work in CMV-positive brain tumours supports this oncomodulatory model. For example, CMV-associated changes have been linked to shifts towards more aggressive transcriptional states in glioblastoma, consistent with viral modulation of tumour programmes in the absence of classic lytic replication^50^. Separately, CMV gene activity has been implicated as a contributor to inflammatory pathway activation within the medulloblastoma microenvironment, suggesting that viral signals can intersect with tumour-associated inflammatory circuits and immune programmes^51^. More broadly, recent syntheses of the “brain-tumour virome” literature emphasize that neurotropic viruses may influence tumour progression primarily through immune modulation, antigen presentation effects and microenvironmental rewiring rather than canonical viral oncogenesis, motivating focused investigation of which viral signals are biologically active in situ and how they interface with tumour–glia–immune communication^52–55^.

A key challenge is causal attribution: detected viral sequences may reflect contamination, bystander (passenger) infection, or compartment-restricted latency rather than biologically meaningful activity; accordingly, onco-modulatory claims should be supported by orthogonal evidence of in situ activity (viral transcripts/proteins), spatial/compartment localization and functional testing (genetic or pharmacological perturbation, including antiviral modulation).

Simultaneously, systemic stress hormones and neuroimmune pathways provide top–down regulation over immune tone and glial function^56–58^. A recent mechanistic study showed that chronic stress increased metastatic outgrowth from disseminated cancer cells by approximately 2–4-fold in mice and remodelled the metastatic microenvironment, including reduced T cell infiltration, increased neutrophil infiltration and stress-hormone-driven induction of neutrophil extracellular traps, with neutrophil depletion abolishing the stress-induced metastatic phenotype^23^. In parallel, emerging work supports direct immunosuppressive effects of the stress–glucocorticoid axis on T cells, including stress-associated induction of tumour-infiltrating CD8⁺ T cell dysfunction in in vivo contexts^59^. Together with broader neuroimmune-crosstalk frameworks, these findings support a model in which extracranial physiology, via endocrine and neural pathways, can reweight immune and stromal states and modulate the stability of intracranial metastatic ecosystems^56^.

Together, these inputs couple extracranial physiology with intracranial tumour progression, effectively embedding brain metastasis within whole-organism regulatory circuits.

## Technologies enabling multiscale systems modelling of brain metastatic communication

Recent advances in experimental and computational technologies have enabled systematic interrogation of communication processes across molecular, cellular, spatial and systemic scales. Rather than increasing resolution alone, these approaches allow reconstruction of interaction networks that underlie metastatic niche formation and maintenance. When integrated across scales, these technologies make it possible to identify emergent properties of metastatic ecosystems and to prioritize communication hubs that sustain tumour-permissive states.

### Spatial transcriptomics and multiplex imaging: where are circuits and microdomains?

Spatially resolved technologies, including spatial transcriptomics, multiplex immunofluorescence and imaging-based proteomics, preserve tissue architecture and enable direct mapping of signalling interactions within metastatic lesions. Spatial analyses have demonstrated that tumour-microenvironment communication is spatially organized rather than uniformly distributed, with signalling interactions enriched at tumour–brain interfaces and perivascular regions where tumour cells, astrocytes, immune populations and vascular elements form structured microdomains. Early mechanistic work demonstrated that metastatic cells preferentially localize to vascular niches and engage reactive astrocytes through proximity-dependent signalling, enabling survival through suppression of plasmin-mediated tumour-cell elimination and establishment of permissive colonization sites^40^. More recent spatial and architectural analyses further showed that brain metastases adopt distinct spatial growth patterns characterized by region-specific microenvironments, in which tumour architecture and local cellular composition co-evolve to support metastatic persistence and immune modulation^44^.

A key advance is that multiplex imaging enables spatial organization to be represented as discrete, testable microenvironmental states. For example, imaging mass cytometry profiling of primary and metastatic brain tumours resolved recurrent spatial cellular neighbourhoods and identified neighbourhood configurations associated with clinical outcome, demonstrating that tissue-level immune architecture is not merely descriptive but can be linked to prognosis and mechanistic hypotheses about local immune regulation^60^.

Consistent with these observations, a pan-cancer single-cell atlas of human brain metastases demonstrated spatial segregation of immune and glial activation states, with immunosuppressive myeloid populations and reactive astrocyte programmes enriched at tumour–brain interfaces and distinct proliferative and metabolic tumour programmes localized within intratumoural regions^17^. Similarly, integrated single-cell and spatial genomic analysis of non-small-cell lung-cancer brain metastases revealed that tumour transcriptional states and immune composition vary systematically across spatial niches, with interface regions enriched for inflammatory and immune-regulatory signalling programmes and tumour cores displaying proliferative and stress-adaptation signatures, indicating that local microenvironmental context shapes tumour evolution and signalling dependencies^33^. Together, these studies demonstrate that proximity-dependent interactions generate localized signalling feedback loops that cannot be inferred from dissociated datasets alone and that spatial organization itself contributes to stabilization of metastatic niches and therapeutic resistance. Spatial profiling therefore defines where communication circuits occur and provides the tissue-level context required to interpret cellular and molecular measurements.

Emerging lineage-aware spatial proteomics further adds causal structure by linking clonal organization to microenvironmental architecture. In xenograft models profiled with epitope barcoding combined with multiplexed ion beam imaging, mixed clones showed emergent spatial behaviours including preferential expansion of clonal patches, and a minority PTEN-deficient subclone increased the patch size of PTEN–wild-type tumour cells, providing direct evidence that subclonal composition can non-autonomously reshape local tumour architecture^61^.

### Single-cell multiomics profiling: what are state transitions and regulators?

Single-cell transcriptomic and multiomics platforms enable high-resolution characterization of cellular heterogeneity and dynamic-state transitions within brain metastases. Single-cell analyses have revealed that immune and glial populations exist along activation continua rather than discrete phenotypes, with transcriptional programmes shaped by local microenvironmental signals rather than lineage identity alone^37^. In particular, single-cell profiling demonstrated that tumour-associated macrophages and microglia occupy intermediate activation states characterized by co-expression of inflammatory and immunosuppressive programmes, indicating that immune function within brain tumours is regulated through continuous state transitions rather than binary polarization.

More recent single-cell atlases of human brain metastases further showed that astrocytes and myeloid populations adopt context-dependent transcriptional programmes linked to immune modulation, metabolic adaptation and stress-response signalling, with regulatory networks differing across tumour types but converging on shared immune-suppressive and tissue-remodelling pathways^17^. Integrated single-cell and genomic analyses of non-small-cell lung-cancer brain metastases similarly identified multiple tumour transcriptional states associated with proliferative, hypoxic and stress-adapted programmes, alongside coordinated changes in surrounding immune-cell states, suggesting that tumour and microenvironmental transitions occur in a coupled manner rather than independently^33^. Consistent with this framework, single-cell transcriptomic profiling of melanoma brain metastases identified distinct tumour-cell subpopulations associated with neural-like and stress-adaptive transcriptional programmes, implicating lineage plasticity and microenvironmental adaptation as drivers of metastatic progression and highlighting transcriptional regulators linked to invasive and survival phenotypes^15^.

Importantly, recent single-cell analyses have also demonstrated that endothelial and mural cell populations actively regulate immune state transitions through expression of immune-modulatory ligands, chemokines and antigen-presentation regulators, indicating that vascular-associated stromal compartments function as upstream regulators of immune tone rather than passive structural components within metastatic lesions^31,32^. Complementing these molecular and cellular observations, mechanobiological studies have shown that physical properties of the vascular microenvironment also influence metastatic behaviour, with differential stiffness between brain vasculature and surrounding parenchyma promoting vessel co-option and invasive adaptation of metastatic cells, indicating that biomechanical cues contribute to the regulation of tumour-cell states alongside molecular signalling inputs^42^. Integration of transcriptomic, epigenomic and proteomic measurements, therefore, enables inference of upstream regulators governing transitions between tumour-restrictive and tumour-permissive ecosystem states, identifying transcriptional and signalling programmes that implement intercellular communication. However, in isolation, dissociation-based approaches cannot determine where or how these interactions occur within tissue architecture, underscoring the need for integration with spatially resolved analyses.

### Ligand–receptor inference: what are candidate communication edges?

Computational ligand–receptor inference frameworks translate single-cell datasets into candidate signalling interactions between cell populations by systematically mapping expressed ligands in one cell population to cognate receptors in another. These approaches enable reconstruction of potential communication edges linking tumour cells with immune, glial and stromal compartments through cytokines, chemokines, growth factors and neurotransmitter-associated signalling pathways. Application of ligand–receptor analysis to brain tumours and brain metastases has revealed extensive bidirectional signalling between tumour cells and astrocytes, myeloid populations and vascular-associated stromal cells, identifying signalling axes that coordinate immune suppression, metabolic adaptation and tissue remodelling across multiple cellular compartments. For example, integration of single-cell datasets with communication inference frameworks has identified chemokine-mediated signalling networks and inflammatory feedback loops linking tumour-associated myeloid cells, astrocytes and tumour cells, suggesting that immune-regulatory programmes emerge from coordinated intercellular signalling rather than from intrinsic cellular states alone^31,37^.

Mechanistic studies further illustrate how such inferred communication edges operate in vivo, including astrocyte-derived TIMP1 signalling that suppresses local CD8⁺ T cell activity and promotes immune evasion within metastatic niches^28^, astrocyte-induced Cdk5 activation in tumour cells leading to downregulation of MHC-I expression and reduced immune recognition^29^, and reciprocal signalling between astrocytes and innate immune cells through lipocalin-2 that reinforces neuroinflammatory and tumour-supportive states^30^. More recent studies combining single-cell and spatial analyses further demonstrated that restricting inferred ligand–receptor interactions to spatially adjacent cell populations substantially improves biological interpretability by eliminating implausible long-range interactions and highlighting niche-specific signalling circuits operating at tumour–brain interfaces^17,33^. Consistent with this framework, spatial cross-talk modelling integrating single-cell and spatial datasets identified CCL4–CCR5-mediated signalling between glial populations as a key communication axis regulating immune modulation and metastatic progression, illustrating how spatially constrained ligand–receptor inference can reveal higher-order communication circuits that are not apparent from transcriptional analysis alone^16^. Incorporation of spatial information therefore reduces false-positive interactions and enables identification of signalling exchanges that are more likely to operate in vivo. Ligand–receptor analysis provides a hypothesis-generating layer that connects cellular states to molecular communication pathways, defining candidate communication edges that can subsequently be prioritized through network modelling and experimental perturbation.

A parallel methodological advance is to prioritize candidate ligands by their predicted downstream impact on receiver-cell gene programmes, rather than by ligand–receptor co-expression alone. NicheNet integrates ligand–receptor relationships with intracellular signalling and gene regulatory networks to link candidate ligands to predicted target genes, enabling selection of interactions whose inferred regulatory consequences match observed receiver-cell transcriptional changes^62^.

### Graph and network modelling: what are hubs and feedback loops?

Network-based modelling approaches integrate inferred communication edges into higher-order interaction maps that capture system-level behaviour across tumour and microenvironmental compartments. Although ligand–receptor inference identifies candidate signalling interactions, graph-based and systems-level analyses enable prioritization of nodes and edges that exert disproportionate influence on network stability, thereby identifying communication hubs and feedback loops that coordinate multicellular responses. In brain metastasis and related tumour systems, these approaches have demonstrated that immune suppression and metastatic persistence frequently arise from interconnected signalling circuits rather than single dominant pathways, with astrocyte–immune, vascular–immune and tumour–stromal interactions forming reinforcing feedback structures that stabilize tumour-permissive ecosystem states^31,37^.

Early computational frameworks for modelling cell–cell communication established the foundation for network-based approaches by integrating ligand–receptor interactions with downstream signalling pathways to reconstruct tumour–stroma communication circuits. Transcriptome-based modelling demonstrated that inferred paracrine and autocrine signalling interactions could be organized into functional communication networks capable of predicting experimentally validated signalling relationships, establishing the principle that intercellular communication can be modelled as an integrated signalling system rather than as isolated pathways^63^. Building on this foundation, platforms such as CCCExplorer (https://github.com/methodistsmab/CCCExplorer)^64^ extend communication inference by linking receptor activation to downstream transcriptional regulators, enabling reconstruction of signalling flow across cellular compartments. Spatially constrained communication modelling approaches such as S2C2 (https://github.com/methodistsmab/S2C2)^65^ further integrate transcriptional states with spatial adjacency and signalling topology to infer continuous intracellular signal propagation from intercellular inputs to regulatory outputs, allowing identification of communication hubs that emerge from convergence of multiple upstream signals onto shared regulatory nodes.

Recent advances in artificial intelligence and machine learning further extend network modelling by enabling integration of heterogeneous datasets and identification of higher-order regulatory patterns across scales. Foundation models developed for single-cell and spatial omics, including transformer-based architectures such as scGPT^66^, demonstrate that large-scale learning across multimodal datasets can capture relationships between cellular states, regulatory programmes and spatial organization, enabling context-aware inference of system-level communication networks and candidate regulatory hubs. Recent work further illustrates how graph-based and multimodal learning frameworks integrate transcriptomic, spatial and imaging-derived features to reconstruct cell–cell interaction structures and infer emergent regulatory modules that are not detectable within individual modalities alone. These approaches leverage graph neural networks and representation learning to model neighbourhood-dependent signalling, predict functional cell–cell interactions, and prioritize network components associated with disease progression or therapeutic response^67–70^. Collectively, these developments position AI-driven modelling as a critical computational layer linking multiscale data integration to mechanistic hypothesis generation and experimental prioritization.

Graph models are also beginning to operationalize relay communication in situ. CellNEST uses attention over spatial neighbourhood graphs to detect multi-step relay-like communication patterns and, across diverse tissues, reports recovery of lymph-node T cell homing signals and identification of aggressive communication programmes in solid tumours, illustrating how network structure can prioritize higher-order signalling motifs beyond single ligand–receptor edges^71^. Complementary frameworks are converging on unified, knowledge-guided analysis of coordinated inter- and intracellular signalling. LIANA+ provides a scalable framework built around a rich knowledge base to decode coordinated inter- and intracellular signalling events from multi-condition datasets in both single-cell and spatially resolved data^72^.

Importantly, communication hubs identified through graph-based and AI-assisted modelling frequently represent nodes that integrate signals across multiple biological scales, linking molecular signalling, cellular-state transitions, spatial organization and systemic modulation. Network modelling therefore provides a principled framework for prioritizing therapeutic targets based on system-level impact rather than on individual pathway dependency, establishing the conceptual bridge between communication inference and experimental perturbation strategies aimed at destabilizing tumour-supportive ecosystems.

### Perturbation-based validation: which hubs are causal?

Experimental perturbation strategies are required to distinguish causal communication hubs from correlative interactions identified through computational inference. Genetic perturbation, pharmacological inhibition and co-culture or organoid-based systems enable functional testing of predicted signalling edges and network nodes by directly assessing their impact on tumour-microenvironment communication. Early mechanistic studies demonstrated that disruption of astrocyte–tumour communication through inhibition of gap junction signalling impaired metastatic survival by altering inflammatory signalling across multiple cell types rather than targeting tumour-intrinsic pathways alone, providing one of the first demonstrations that perturbation of intercellular communication can destabilize the metastatic niche^27^.

More recent studies have extended this principle by demonstrating that perturbation of astrocyte- and immune-mediated signalling axes produces coordinated ecosystem-level effects. For example, inhibition of astrocyte-derived TIMP1 signalling restores CD8⁺ T cell activity and reduces local immunosuppression within brain metastases^28^, whereas disruption of astrocyte-induced Cdk5 signalling in tumour cells restores antigen presentation and enhances immune recognition^29^. Similarly, perturbation of inflammatory feedback loops between astrocytes and innate immune populations through lipocalin-2 signalling alters neuroinflammatory states and limits metastatic progression^30^. These findings collectively demonstrate that perturbation at the level of communication hubs frequently induces coordinated changes across multiple cellular compartments, consistent with the prediction that metastatic stability arises from network organization rather than from single signalling pathways.

Emerging perturbation frameworks further enable systematic testing of network predictions by combining genetic or pharmacological perturbations with single-cell and spatial readouts, allowing direct measurement of how disruption of candidate hubs reshapes cellular states and communication circuits. Recent in vivo Perturb-seq approaches demonstrate that pooled CRISPR perturbations coupled with single-cell transcriptomic profiling can resolve how genetic perturbations propagate across tumour and microenvironmental compartments, revealing ecosystem-level responses that extend beyond cell-autonomous effects^73,74^. Spatially resolved perturbation platforms further extend this framework by preserving tissue architecture during CRISPR screening, enabling direct assessment of how genetic perturbations alter spatial organization and intercellular signaling within intact tissue environments^75^. CRISPR-based perturbation approaches and perturbation-coupled single-cell profiling therefore increasingly allow causal validation of predicted regulatory nodes by linking gene disruption to downstream changes in intercellular signalling and ecosystem composition. Within a multiscale framework, such perturbation strategies provide the critical experimental layer that converts inferred communication networks into mechanistically validated therapeutic targets and contributes to emerging efforts to construct causal perturbation atlases of cellular systems^76,77^.

Human-relevant organoid and assembloid systems provide a complementary route for testing niche mechanisms under controlled spatial architectures. In small-cell lung-cancer models, tumour–brain organoid assembloids and intracranial experiments identified a mechanistic tumour-to-astrocyte-to-tumour loop in which tumour-secreted Reelin recruits astrocytes and astrocyte-derived SERPINE1 supports tumour growth, directly linking a specific intercellular axis to metastatic outgrowth^36^.

Together, these technologies enable a transition from descriptive characterization of brain metastasis toward predictive modelling of metastatic ecosystems. By integrating spatial organization, cellular state transitions, molecular signalling and functional perturbation, multiscale systems modelling provides a framework for identifying vulnerabilities that emerge from communication networks and feedback loops sustaining tumour-permissive niches.

## Translational implications and future directions

Reframing brain metastasis as a disorder of multiscale communication fundamentally alters therapeutic strategy. Rather than focusing exclusively on tumour-intrinsic vulnerabilities, this perspective emphasizes disruption of the communication networks that stabilize metastatic niches. Importantly, many persistent clinical challenges in brain metastasis, including variable immunotherapy response, intracranial resistance despite extracranial control, and poor predictive performance of single biomarkers, may reflect failure to account for ecosystem-level regulation. A systems framework provides a basis for addressing these unresolved clinical uncertainties.

### Network-based therapeutic strategies

#### Why do immune checkpoint inhibitors frequently fail in symptomatic brain metastasis despite activity in extracranial disease?

Accumulating evidence suggests that therapeutic resistance often arises from local immune suppression mediated by tumour–glial and stromal communication rather than intrinsic resistance of tumour cells to immune attack. Astrocyte-driven signalling programmes, including TIMP1-mediated suppression of CD8⁺ T cell activity^28^ and Cdk5-dependent reduction of tumour antigen presentation^29^, demonstrate how local communication networks can limit immune recognition even when systemic immunity is intact. Targeting communication hubs at the tumour–glial or immune–stromal interface therefore represents a strategy for restoring immune accessibility by destabilizing niche-level immunosuppressive states rather than directly enhancing tumour cytotoxicity. Such approaches support a shift from pathway inhibition toward network destabilization as a therapeutic objective.

Complementing this concept, spatial cross-talk modelling and experimental validation have identified chemokine-mediated glia–glia communication as another actionable hub within the metastatic niche. Computational reconstruction of tumour–glial signalling networks using CCCExplorer identified a restricted set of astrocyte-derived signalling pathways enriched during brain metastasis progression, with CCL4–CCR5 signalling emerging as a dominant axis coordinating astrocyte activation and downstream tumour signalling. Functional studies demonstrated that tumour-conditioned astrocytes increased CCL4 secretion and induced CCR1/CCR5-dependent activation of PDGFRA-, TEK- and FGFR-associated signalling pathways in tumour cells, promoting Ras and gap junction signalling activation. Genetic disruption of CCL4 or pharmacological inhibition of CCR5 attenuated downstream signalling and reduced metastatic progression in vivo. Notably, the clinically approved CCR5 antagonist maraviroc prevented brain metastasis formation in early treatment settings and reduced established tumour burden by approximately 62% without direct tumour cytotoxicity, consistent with a communication-hub mechanism rather than tumour-intrinsic targeting^16^. Spatial transcriptomic analyses further demonstrated reduced activation of CCR5-associated signalling specifically within tumour-adjacent microdomains following treatment, supporting the concept that therapeutic benefit arises from disruption of localized communication circuits.

Together with earlier demonstrations that astrocyte–tumour gap junction signalling sustains metastatic survival through inflammatory signalling transfer^27^, these studies support a network-based therapeutic paradigm in which partial perturbation of high-leverage communication hubs can shift the metastatic ecosystem away from tumour-permissive states. Such strategies suggest that targeting intercellular signalling interfaces, rather than individual oncogenic pathways, may represent a scalable approach to overcoming intracranial therapeutic resistance.

### Drug repurposing and combination approaches

#### Why does intracranial disease frequently progress despite systemic control with targeted therapy or immunotherapy?

The systems framework suggests that intracranial resistance often reflects adaptation of the metastatic ecosystem rather than acquisition of tumour-intrinsic resistance mutations. Agents originally developed for antiviral, neuropsychiatric or immunomodulatory indications may exert therapeutic benefit by reprogramming communication networks that sustain metastatic stability. For example, inhibition of CCR5 signalling disrupts glia-mediated signalling without direct tumour cytotoxicity^16^, illustrating how targeting intercellular communication can alter niche behaviour. Combining such agents with existing targeted therapies or immune checkpoint blockade offers a rational approach to overcoming intracranial resistance, particularly when guided by network modelling rather than empirical trial-and-error.

### Predictive and patient-specific modelling

#### Why do single biomarkers fail to reliably predict therapeutic response in brain metastasis?

Single biomarkers incompletely capture the spatial and cellular heterogeneity that governs metastatic behaviour. Increasing evidence indicates that therapeutic response in brain metastasis is shaped by ecosystem-level organization involving tumour-cell state, immune composition, vascular signalling and glial interactions rather than tumour genotype alone. A recent pan-cancer single-cell atlas integrating 108 brain metastases and 111 matched primary tumours demonstrated convergence towards shared metastatic programmes across tumour types, including increased chromosomal instability, proliferative and angiogenic signalling, and neural-like transcriptional programmes, with immunosuppressive stromal and myeloid ecosystems associated with poorer clinical outcomes^17^. These findings support the concept that clinically relevant patient subgroups may be defined by ecosystem configuration rather than lineage-specific mutations.

Consistent with this framework, multimodal profiling of treatment-naive non-small-cell lung-cancer brain metastases integrating single-nucleus RNA sequencing, spatial transcriptomics and whole-genome sequencing demonstrated that chromosomal instability represents a defining feature of metastatic progression and is associated with enrichment of neural-like tumour-cell states within intracranial lesions^33^. Importantly, tumour populations high in chromosomal instability were detectable in primary tumours but selectively expanded in brain metastases, suggesting that metastatic competence arises from pre-existing cellular states shaped by the brain microenvironment. Spatial analyses further confirmed that these neural-like tumour populations co-localize with distinct microenvironmental niches, reinforcing the need for predictive models that integrate tumour-intrinsic programmes with tissue-level organization.

Additional studies integrating spatial and single-cell datasets demonstrate that vascular and stromal signalling programmes further influence therapeutic response. Endothelial and mural cell atlases have identified immune-regulatory vascular states that modulate immune infiltration and treatment sensitivity, highlighting vascular-immune communication as an additional predictive layer in brain metastasis biology^31,32^. Together, these studies suggest that predictive modelling must incorporate both tumour-cell state and microenvironmental architecture to accurately capture therapeutic vulnerability.

Artificial-intelligence and machine-learning approaches increasingly enable integration of transcriptional, spatial and imaging data to identify emergent interaction patterns predictive of disease progression or therapeutic response. Incorporating multiscale features such as spatial immune organization, glial signalling states and systemic modulators into predictive frameworks supports personalized intervention strategies and adaptive trial designs in which therapies are selected based on dominant communication hubs operating within individual metastatic ecosystems.

### Future directions

Key challenges remain in translating multiscale frameworks into clinical practice, including standardization of spatial and computational pipelines, reproducibility across platforms and institutions, and integration of systemic physiological variables such as immune status, stress signalling and treatment history into modelling frameworks. Equally important is the need to convert complex multiscale analyses into clinically interpretable tools that can guide therapeutic decisions and trial design. Addressing these challenges will require sustained interdisciplinary collaboration across oncology, neuroscience, immunology, engineering and computational biology, as well as prospective studies linking multiscale measurements to patient outcomes.

**Fig. 2:**
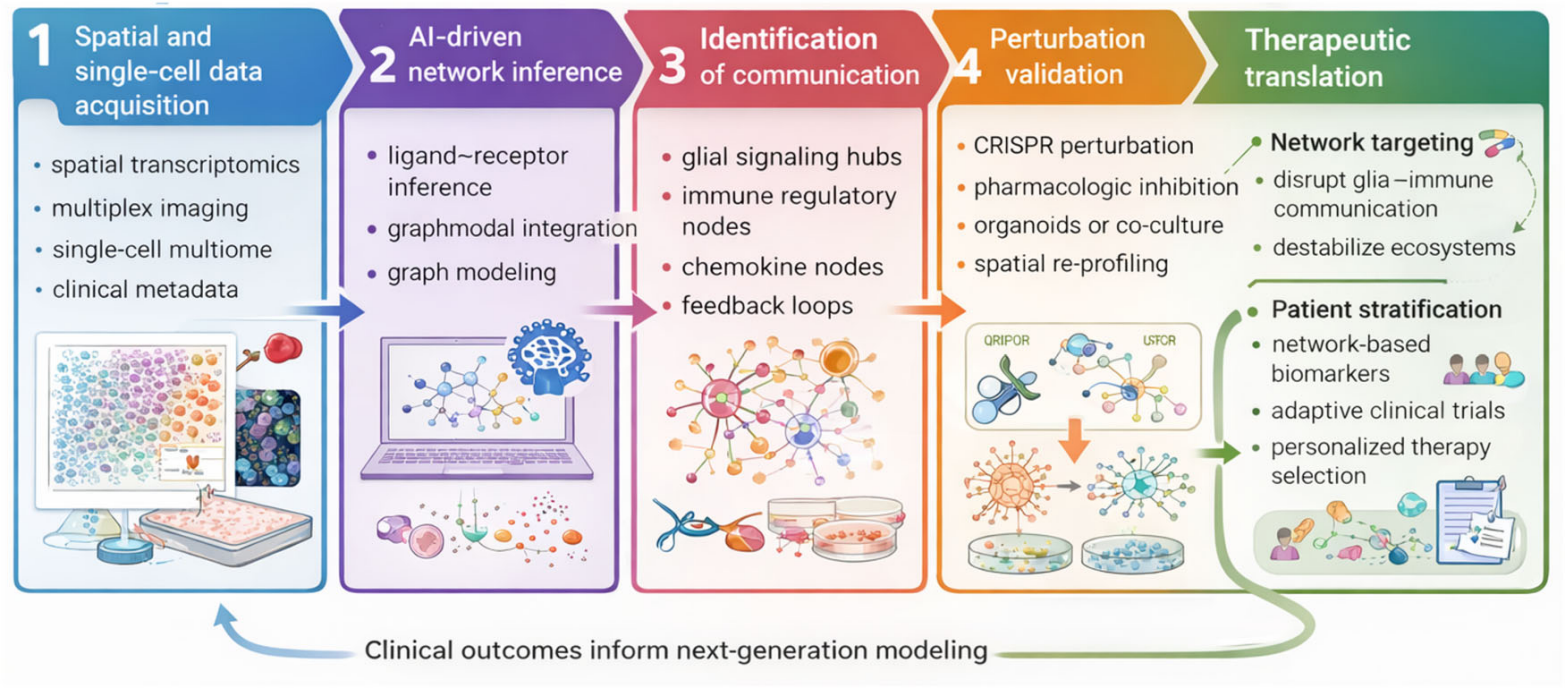
Translational framework linking multiscale network discovery to therapy. Multiscale profiling approaches, including spatial transcriptomics, multiplex imaging and single-cell analyses, generate high-resolution molecular and spatial data from brain metastases. Integration of these datasets using artificial intelligence and network modelling enables reconstruction of intercellular communication networks and identification of candidate communication hubs. Genetic and pharmacological perturbation strategies are then used to distinguish causal regulatory nodes from correlative interactions. Validated hubs inform therapeutic translation through network-based targeting, drug repurposing and rational combination strategies, and incorporation of network features into patient stratification and adaptive clinical trial design. This framework illustrates how multiscale modelling connects biological discovery to predictive and actionable therapeutic development. Created using BioRender.

Despite these challenges, the rapid convergence of spatial profiling, single-cell technologies and artificial intelligence-enabled modelling positions the field to move beyond descriptive characterization towards predictive and interventional systems biology. By identifying communication hubs and ecosystem states that govern metastatic stability, future work may enable rational disruption of tumour-supportive networks and establish a new generation of mechanistically guided therapies for brain metastasis (Fig. 2).

## Conclusions

Brain metastasis remains a major clinical challenge because its behaviour cannot be explained solely by tumour-intrinsic biology. The evidence summarized in this Review supports a view in which metastatic progression and therapeutic resistance emerge from coordinated interactions across molecular, cellular, spatial and systemic levels. These interactions generate reinforcing feedback loops and stable tumour-permissive ecosystems that cannot be effectively addressed through single-pathway (single-target) or tumour-centred strategies alone.

Recent advances in spatial profiling, single-cell and multimodal omics, and computational modelling now make it possible to characterize these interactions directly and to define communication networks that govern metastatic stability. As these technologies converge, they provide a framework for identifying actionable communication hubs, refining patient stratification, and guiding rational therapeutic combinations that target tumour–microenvironment coupling rather than isolated pathways. Critically, translation will require moving beyond association: candidate hubs must be prioritized using cross-cohort replication, orthogonal validation and perturbation-based evidence of functional necessity and sufficiency.

Future progress will depend on translating multiscale biological insight into clinically interpretable models and deployable intervention strategies. Viewing brain metastasis through a systems-level lens shifts emphasis from eliminating individual components towards disrupting the network mechanisms that sustain disease and re-stabilizing anti-tumour ecosystem states. This transition creates an opportunity to move from descriptive understanding towards predictive, patient-specific and interventional approaches, including biomarker-linked adaptive trials, capable of improving durable intracranial control.

### Perspective: Open questions in multiscale communication modelling of brain metastasis

Despite growing recognition that brain metastasis is governed by multiscale communication networks, several fundamental questions remain unresolved. How do glial communication states evolve over time during metastatic seeding, dormancy and therapeutic intervention, and are there irreversible “commitment points” that lock niches into tumour-permissive attractor states? To what extent do latent viral infection, stress signalling and neurotransmitter rewiring converge on shared glial and immune hubs, versus operating through parallel, context-dependent circuits? Can spatial communication signatures be distilled into predictive biomarkers that stratify patients and guide combination therapies in real time? Finally, how can computational models be standardized, validated and translated into clinically actionable tools without sacrificing biological nuance? Addressing these questions will require longitudinal, spatially resolved profiling coupled with perturbation-informed systems modelling, but doing so holds the promise of transforming brain metastasis from a therapeutically intractable condition into a predictable and targetable communication disorder.
